# Computing mutual similarity of 3D human faces in nearly linear time

**DOI:** 10.1371/journal.pone.0329489

**Published:** 2025-08-04

**Authors:** Radek Ošlejšek, Petra Urbanová, Jiří Sochor

**Affiliations:** 1 Faculty of Informatics, Masaryk University, Brno, Czech Republic; 2 Faculty of Science, Masaryk University, Brno, Czech Republic; Tongji University, CHINA

## Abstract

Using three-dimensional scans of human faces has become an emerging technique in studies of human variation, where the quantitative assessment of facial similarity complements the measurement of other somatic traits. While the algorithms for automated registration (geometrical alignment) and similarity measurement of two facial scans are well-known and used in practice, their direct application for batch processing is limited due to computational requirements. The batch N:N analysis, where all pairs of scans in a dataset must be mutually registered and compared, introduces quadratic complexity with computation times reaching hours even for relatively small datasets, making it practically unusable. This paper presents a rapid and accurate approach with nearly linear time complexity. Our solution utilizes properties of facial scan geometry to optimize individual steps. Moreover, the algorithm deals with possible holes and other artifacts in polygonal meshes automatically. Experiments demonstrate that the proposed solution is very fast and sufficiently accurate compared to a precise quadratic-time baseline approach.

## Introduction

Identification and allocation of individuals into groups are fundamental methods in biological and physical anthropology used to explore and understand human biological variation across and within populations [1]. It typically involves the quantitative assessment of similarities and dissimilarities in somatic traits (anthropometric characteristics, physical appearance, cranial and dental features, trait frequencies, etc.).

The number of (dis)similarities determines biological distance or *biodistance*. In the past, it was assumed that biodistances were underlying reflections of genetic relationships [2]. However, contemporary understanding reveals that these assumptions are more intricate. Nonetheless, biological distance analysis has proven valuable in illuminating evolutionary processes [3], migration patterns [4], population diversity [5], and historical factors influencing human variation over time and across regions [6,7].

Biological distance can be expressed in multiple ways. Following rapid development over the past two decades, 3D faces can now be created easily using a variety of three-dimensional imaging modalities, ranging from medical imaging to laboratory equipment to personal devices such as tablets or cellphones. However, as the data samples differ in quality and format of geometry encoding, e.g., depth images, point clouds, or meshes, they are not equally suitable for biological anthropology [8].

This paper deals with biodistances computed for *stereophotogrammetry data*. Devices based on stereophotogrammetry are widely used for capturing the physical appearance of a person. The data consists of a 3D facial geometry in the form of a 3D mesh and facial colors in the form of a texture (2D image). Both components have been subject to a variety of comparison and/or identification efforts separately [9]. Still, it is the combination of facial image and 3D shape that makes facial analysis more feasible and efficient [10], making this kind of data popular in the respective application domains. Although our method strictly relies only on the geometrical component, information taken from photo textures can be used for further improvements in the future, e.g., to involve the color of eyes in the similarity measurement.

Techniques of quantitative description and comparison of mesh models can be divided into two categories. Descriptor-based biodistance approaches utilize local features, e.g., curvature, to deal with special cases like pose-invariant 3D face recognition, expression—invariant recognition, or incomplete data [11,12]. These methods are independent of the position of 3D models in space. In contrast, registration-based methods rely on the position of faces in space. The similarity methods assume that the analyzed 3D models are mutually aligned – so-called registered.

Despite the fact that the registration is computationally intensive and prone to imperfections that can affect the measurement, registration-based methods remain popular among anthropologists due to their high precision and the explainability of expert decisions supported by a data-driven approach. Therefore, our method uses results achieved in the registration-based biodistance measurement of 3D facial pairs to accelerate the similarity measurement of all faces in a population.

### Problem statement

**Fig 1:**
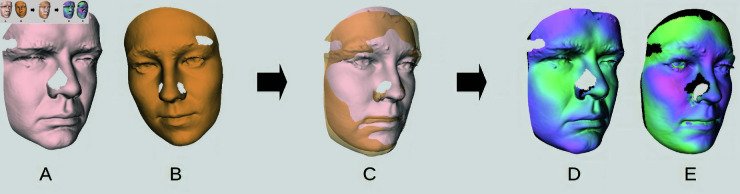
Registration and distance measurement of two faces. Views A and B capture original 3D scans with holes, view C faces after registration, view D relative distance from the first face to the second one, and view E vice versa. Black areas denote automatically detected non-overlapping surfaces that are omitted from the measurement.

For a single pair of faces, registration-based similarity measurement consists of two key steps suggested in Fig 1. First, the two faces *A* and *B* are registered (precisely aligned in space with each other (preview *C* in Fig 1). Then, the distance between their surfaces is measured, with the distances shown as color heatmaps in views E and F.

Given a set of 3D facial scans, our goal is to register and measure all pairs in the dataset, aiming to compute the mutual (dis)similarity of all faces. We use the terms *N:N* or *batch* in the remainder of the paper to express these bulk operations, e.g., *N:N registration* means the mutual registration of all faces.

The precision of registration can significantly affect the results of the similarity measurement. Therefore, the best results can be achieved if all pairs of faces are registered and measured independently of other faces in the dataset. However, such a pairwise approach is extremely inefficient due to the quadratic complexity. Computation times exceed hours or days in the case of hundreds of faces, making this approach precise but impractical. However, to the best of our knowledge, this time-consuming approach was the only method used so far by anthropologists to analyze dissimilarities in a collection of 3D facial scans.

The idea of accelerating the registration phase lies in aligning all faces at once, i.e., to optimize the position of all faces relative to all other faces in the dataset. However, the price we pay for this optimization may be lower accuracy because the position of any two faces can be considered a compromise reflecting not only the second face but the shape of all other faces from the dataset.

Generalized Procrustes Analysis (GPA) [13] represents a popular method often used in robotics and image reconstruction to mutually register many 3D objects at once in linear time. However, GPA suffers from a strong assumption – the existence of an explicit pairing between corresponding points from analyzed objects. Facial scans obtained from stereophotogrammetry consist of polygonal meshes, and, therefore, they provide no such correspondence, making the GPA directly unusable.

In the anthropological domain, the correspondence of analyzed subjects could be determined through a process of landmark detection, where discrete anthropometric points, i.e., landmarks with an established biological or geometric meaning like the inner eye corner, are often used in facial analysis [14]. However, their extraction from 3D models with sufficient accuracy is laborious and difficult to automate. This is why many existing auto-detection techniques deal with only a few selected landmarks that are easy to detect [15–18]. Using GPA with only this limited selection of automatically extracted landmarks would essentially prioritize corresponding facial areas at the expense of areas for which landmarks were not detected. For these reasons, we aim to find a solution to batch registration that deals with polygonal meshes directly.

Popular ICP-based registration methods [19–21] overcome the correspondence requirement by computing 3D transformations that directly minimize the distance between vertices of 3D meshes. However, they only address the pairwise problem. Its direct usage leads to the already mentioned suboptimal pairwise registration running in quadratic time.

Based on these observations, we formulate the first research question:

- **RQ1: How can a set of polygonal 3D facial scans be registered efficiently, yet precisely?** A possible solution is to combine GPA and ICP principles appropriately, carefully addressing robustness and precision.

Processing large polygonal meshes, as to be expected in biodistance analyses, is limited due to low efficiency, regardless of which method is used. Therefore, sub-sampling strategies have to be used in practice to reduce the number of vertices of original objects. However, the strength of sub-sampling can negatively affect the precision of results [22]. Also, the strategy of selecting vertex samples from original 3D models, e.g., random or uniform distribution of samples, can affect both efficiency and precision. These facts lead to the following research question:

- **RQ2: How do the sub-sampling methods affect the efficiency and precision of batch registration?** The goal is to find the best sub-sampling methods and their parameters that provide efficient registration while preserving high precision.

Assuming that 3D models are mutually registered, the computation of the similarity of all pairs of faces in the dataset introduces, again, quadratic complexity. Moreover, the algorithms are computationally intensive. Surfaces must be sufficiently sampled into dense meshes to compute a surface-to-surface deviation precisely. Despite using space partitioning techniques to accelerate face-to-face measurement [23,24], the need to compare all pairs of faces in the dataset prolongs the computation unacceptably. The time requirements are similar to the sub-optimal pairwise registration. The need for the radical optimization of this step leads to the following research question:

- **RQ3: How to measure a mutual similarity of a set of registered 3D facial models efficiently?** Similarly to efficient batch registration, the goal is to achieve linear time complexity of the measurement without losing the precision of measurement.

The results of 3D image acquisition modalities are often prone to errors, such as holes, defective polygons, or smudged textures. The presence of reflective, moist, and hairy areas (such as scalp, body or facial hair, and eyeballs) has been singled out as troublesome for photogrammetry-based 3D reconstructive algorithms [25]. This either leads to gaps and blank regions in the meshes, as the data preprocessing skips the creation of a mesh in sensitive areas to avoid an imperfect result, or a flawed, unreliable geometry is built.

Moreover, unlike many other 3D objects, 3D scans of human faces do not form closed shapes. They resemble a surface shell with scrappy edges into which data errors and noise are inserted during 3D scanning. ICP-based approaches have been shown to perform well in face identification when only processing two similar and uniformly cropped 3D scans [22]. Erroneous bordering parts can significantly affect results when comparing two or more facial scans. Therefore, these areas must be removed during preprocessing, either automatically or manually, which leads to the following research question:

- **RQ4: How to deal with holes and scrappy edges?** The goal is to make the batch registration and similarity measurement robust for the real data. The algorithms have to deal with preprocessing artifacts, i.e., holes, and simultaneously minimize the impact of boundary areas by automatically removing them from the calculations. All that without significantly decreasing efficiency.

### Related work

Many articles are devoted to 3D face identification, i.e., establishing a person’s identity by comparing their physical characteristics with samples stored in a database. Although the identification task differs from the N:N similarity measurement, both research directions share certain principles and geometric approaches. In the latest overview papers related to 3D face recognition [26,27], the authors review 3D face recognition techniques developed in the past decades. Both conventional and deep learning-based methods are discussed.

Zhou *et al*. [11] also provide a comprehensive survey on 3D face recognition techniques, data types, and obstacles. Considering their description of the research field, our solution is limited to dealing with reasonably complete polygonal meshes. Moreover, challenges related to occlusion, head poses, or different facial expressions are beyond the scope of this paper. Li *et al*. [12] categorize 3D face recognition methods using a classification tree. According to this classification, our registration-based algorithmic solution fits the *global feature-based* category and, especially, the *spatial-based* and *geometry domain* sub-categories.

The registration-based similarity of two 3D objects is extensively discussed in the literature. In [28], Castellani and Bartoli summarize key steps, challenges, and optimization techniques. According to them, the precision of registration and distance measurement strongly depends on the ability to filter out noisy parts and outliers. As eliminating non-overlapping surfaces has been shown to increase the accuracy [29,30] of ICP registration in general, we use this technique to prioritize anthropometry-significant facial areas by automatically eliminating noisy marginal parts of 3D facial scans. This technique improves both the precision of the registration and the similarity measurement of two faces.

Jurda and Urbanová [31] utilized root mean square and other descriptive statistical parameters to quantify dissimilarities while categorizing cranial 3D models into sex and ancestral groups. Similarly, Jandová and Urbanová [32] employed identical distance measures to cluster 3D facial shell scans based on the degree of facial deformation linked to archetypal emotions and simulated facial expressions.

When it comes to efficiency, many sub-sampling studies can be found in the literature evaluating the impact of sub-sampling on the acceleration of ICP-based registration [22,33,34]. They cover a random selection of points, uniform space sampling approaches [30], or the selection of significant points based on local features, e.g., curvature [35]. Since the shapes of 3D facial scans exhibit specific geometric properties, we evaluate three selected techniques to determine the optimal balance between efficiency and precision in N:N registration.

The precision and efficiency of the pairwise registration and similarity measurement can be improved by many other techniques, e.g., using 3D shape descriptors [36–38]. However, while these methods enhance face-to-face analysis, they still do not resolve the quadratic complexity of the N:N analysis.

Our approach to addressing the quadratic complexity of batch registration is based on the combination of GPA and ICP. This idea is not new. Toldo *et al*. [39] combine GPA with ICP in their multi-view registration, aiming to automatically reconstruct an object’s shape from 3D scans captured from different angles. Podimor *et al*. [40] describe the registration and similarity measurement of a set of 3D skulls. Their method uses a special cost function and modified Procrustes Distance Metric to tackle the asymmetry of distance computation and achieve the best results. Both papers deal with point clouds. On the contrary, our algorithm benefits from the mesh topology included in the stereophotogrammetry data and the specific geometry of 3D facial scans to combine GPA and standard non-symmetric ICP efficiently yet precisely. Moreover, to the best of our knowledge, our solution that uses the average face produced by the registration step as a gauge for fast indirect distance measurement of individual pairs is unique.

## Methods

The project was approved by the Research Ethics Committee of the Faculty of Science, Masaryk University. All participants involved in the face scanning process provided informed consent. Adult participants signed the consent form themselves, while for minors, consent was obtained from their parents or legal guardians.

### Formal definitions

#### Nearest neighbors of a point.

Given a point in 3D space $v\in {𝑅}^{3}$, we define its nearest neighbors from face *f* as the set of points w∈f with the smallest Euclidean distance to *v*:

near(v,f)={w∈f∣∀u∈f:||v−w||≤||v−u||} (1)

**Fig 2:**
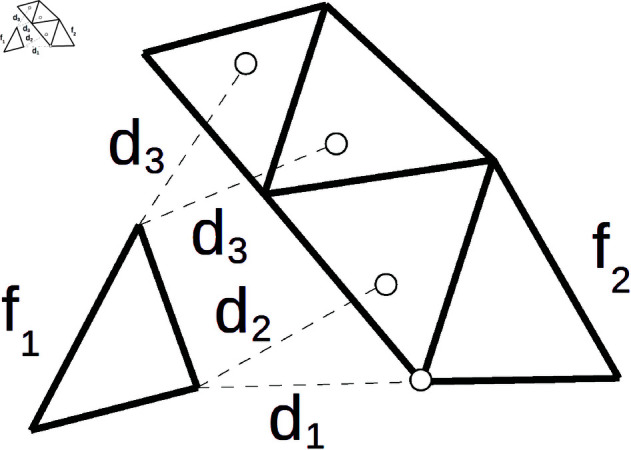
Nearest neighbors. The goal is to find the closest points laying on face *f*_2_ for all vertices of face *f*_1_ laying on *f*_2_. Distance *d*_1_ is obtained if only vertices from *f*_2_ are considered. More precise distance *d*_2_ takes the whole surface of *f*_2_ into account, aiming to find the closest point lying anywhere on the polygonal surface. Distances *d*3 capture a rare situation when two nearest neighbors exist. In this case, the corresponding vertex at *f*_1_ is removed from the calculations.

If the geometry of face *f* is represented by independent 3D points (i.e., a point cloud), then the set includes the closest points from *f*, as shown in Fig 2. However, if triangular meshes are used, then the nearest points can lie anywhere on the polygonal surface of *f*, including the edges and inner parts of triangles.

#### Reduced surface.

Similarity of two faces is computed by inspecting all vertices of one face and finding their nearest neighbors on another face. However, Eq 1 is not deterministic because multiple nearest neighbors can exist (lying at the same distance, as shown in Fig 2, distance *d*_3_). Therefore, we define

$$\bar{f_{i}}(f_{j})=\{v\in f_{i}:|near(v,f_{j})|=1\}$$ (2)

$\bar{f_{i}}(f_{j})$ is reduced surface of *f*_*i*_ consisting of vertices with only one nearest neighbor from *f*_*j*_. We use $\bar{f_{i}}(f_{j})$ instead of *f*_*i*_ in further calculations as this reduction makes the computation a partial function $\bar{f_{i}}(f_{j})\to f_{j}$, then deterministic. For the sake of simplicity, we shorten the notation to

$$\bar{f_{i}}:=\bar{f_{i}}(f_{j})$$ (3)

whenever the context of *f*_*j*_ is clear. It should be noted that excluding vertices with multiple nearest neighbors does not impact the results of the registration or similarity measurement algorithms discussed later. This is due to the negligible probability of encountering multiple nearest neighbors in real 3D facial scans. Our analysis of 500 faces showed that only 0.01% of vertices had multiple nearest neighbors. Therefore, these cases can be considered noise, which is tackled by the approximate registration and measurement anyway.

#### Similarity of one face to another.

The distance-based similarity of face *f*_*i*_ to face *f*_*j*_ is estimated by measuring Euclidean distances between all vertices of $\bar{f_{i}}$ and their nearest neighbors from *f*_*j*_.

#### Mutual similarity of two faces.

For many practical applications, including our batch similarity measurement, a single-value distance indicator is required. It can be obtained by properly combining individual values obtained from the similarity of one face to another. However, the measurement has to be performed in both directions, i.e., $\bar{f_{i}}\to f_{j}$ and $\bar{f_{j}}\to f_{i}$ because the operation is asymmetric. Traditional Hausdorff distance (HD) measurement [41] adopts this principle by finding the maximum gap between two surfaces. However, it makes the measurement sensitive to noise and outliers. In this paper, we use a modified HD function that provides better results for object-matching tasks [42]. This modification averages distances of nearest neighbors instead of finding their supremum. Formally, we define a **distance function** *dist* of two faces *f*_*i*_ and *f*_*j*_ as:

$$dist(f_{i},f_{j})=max(d_{avg}(f_{i},f_{j}),d_{avg}(f_{j},f_{i}))$$ (4)

where $d_{avg}$, referred to as **distance cost function**, computes the average distance of vertices from *f*_*i*_ to their nearest neighbors from *f*_*j*_, excluding vertices with multiple nearest neighbors:

$$d_{avg}(f_{i},f_{j})=\frac{1}{\left|{\bar{f}}_{i}\right|}\sum_{v\in {\bar{f}}_{i}}\|v-near(v,f_{j})\|$$ (5)

#### Registration of one face to another.

Denote *T*(*f*) a face transformed by an affine transformation matrix *T*. ICP-based registration aims to find transformation *T* that aligns a face *f*_*i*_ onto *f*_*j*_ by minimizing the residual distances between *T*(*f*_*i*_) and their nearest neighbors from *f*_*j*_:

$$icp(f_{i},f_{j})=\underset{T}{\min}\;d_{avg}(T(f_{i}),f_{j})$$ (6)

#### Mutual registration of two faces.

The *icp* calculation is also asymmetric. Therefore, the registration of two faces $f_{i},f_{j}$ aims to find the best transformation in both directions. More formally, it can be considered a minimization function that uses *icp* to find an affine transformation *T* of *f*_*i*_ to *f*_*j*_ or vice versa so that the distance cost function $d_{avg}$ of two faces *f*_*i*_ and *f*_*j*_ is minimized.

### Solution to batch registration

Traditional GPA-based registration of objects with explicit pairing consists of four basic steps. Our solution addresses *RQ1* by adopting this process but replacing Procrustes superimposition with ICP.

#### Step 1 – selection of a template face.

**Fig 3:**
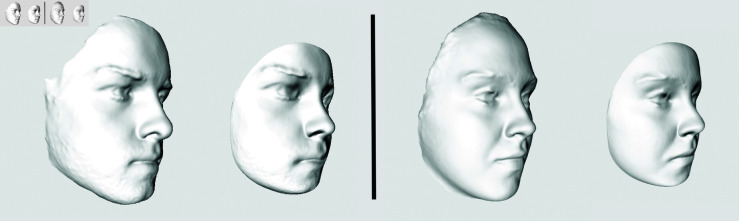
Average faces. They are obtained by transmuting a template face with respect to a collection of 100 other faces (they are not shown). Two different template faces (on the left for each pair) selected from the same data set can produce slightly different average faces (on the right for each pair)).

First, a face from the data set must be chosen and cloned. This face then metamorphoses into an average face and serves as the reference shape to which other faces are registered. The selection of the template face depends on the anthropologist’s decision and can affect the results of the analysis. This is because the average face calculated in step 3 by adapting the vertices of the template face can produce slightly different surfaces for different initial faces, as shown in Fig 3. However, studying this impact on overall results is beyond the scope of this paper. We rely on the expertise of the analyst.

#### Step 2 – ICP-based superimposition.

All faces from the dataset are aligned with the template face using transformation matrices computed by the ICP function (Eq 6). This step replaces standard Procrustes superimposition that requires correspondence between points from surfaces, which is not available for 3D meshes. However, such replacement is not straightforward. The primary limitation is the asymmetry of ICP. In the case of registering only two faces, both directions can be calculated, and the better one is selected. However, in GPA, the registered face is always transformed towards the template face, while the location of the template face remains unchanged. This limitation can introduce a significant error if the asymmetry of ICP is substantial. Podimor *et al*. [40] solve this issue by using a special cost function and modified Procrustes Distance Metric in the GPA algorithm. We show that one-directional registration can be effectively used for 3D facial scans without significant loss of precision if the primary source of asymmetry is mitigated at the geometric level.

#### Step 3 – averaging faces.

This step transforms the template face into the average face, considering the new positions of other faces. Geometrically, the template face metamorphoses into the average of all other faces by moving its vertices toward the centroids of nearest neighbors. Formally, consider a vertex $v\in f_{t}$, where *f*_*t*_ is the current template face. We define its average position $v_{a}$ within the set of faces f∈F as

$$v_{a}(v,F)=v+\frac{1}{\left|F\right|}\sum_{f\in F}v-near(v,f)$$ (7)

Similarly to the distance cost function defined in Eq 5, faces f∈F for which the |near(v,f)|>0 can be skipped. Then, the average face *f*_*a*_ computed from the template face *f*_*t*_ with respect to all faces from *F* is defined as

$$f_{a}=\{v_{a}(v,F)\;|v\in f_{t}\}$$ (8)

#### Step 4: – next iteration.

If the shape of the average face *f*_*a*_ significantly differs from the template face *f*_*t*_, then the template face is replaced with the average face, and the algorithm returns to step 2. Iterative repetition of steps 2 and 3 continuously transmutes the template face selected in step 1 into the average of faces from the dataset that are simultaneously aligned with the average face. The calculation ends when the system stabilizes, i.e., when the change in distances $dist(f_{a},f_{t})$ across iterations falls below a predefined threshold.

Our experiments revealed that three iterations are usually sufficient to get optimal alignment. This observation is consistent with the results published in [40]. Therefore, the entire N:N registration process can be considered to run in linear time with respect to the number of faces.

### Solution to batch measurement

Once the faces are registered with the average face, their mutual similarity could be measured by comparing all pairs of faces from the dataset using the distance function from Eq 4. However, the time requirements are, again, quadratic.

The idea of reducing the complexity of the pairwise approach (and then addressing *RQ3*) lies in measuring the distance between two faces indirectly, involving the already computed average face as a gauge.

A generic formula for the discrete average **indirect distance measure** of faces $f_{i},f_{j}\in F$ via the average face *f*_*a*_ adapts Eqs 4 and 5 as follows:

$$dist_{ind}(f_{a},f_{i},f_{j})=\frac{1}{\left|{\bar{f}}_{a}\right|}\sum_{v\in {\bar{f}}_{a}}d_{ind}(v,f_{i},f_{j})$$ (9)

where ${\bar{f}}_{a}$ denotes for *f*_*a*_ restricted to only vertices with exactly one nearest neighbor from *f*_*i*_ and *f*_*j*_.

**Fig 4:**
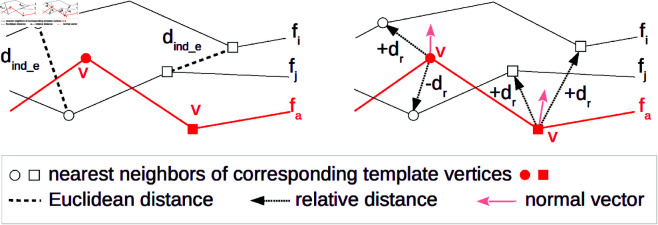
Indirect measurements visualized in 2D. Euclidean distance of nearest neighbors (left) vs. subtraction of their relative distances from the template vertex *v* (right).

The aim of the *d*_*ind*_ function is to approximate the computation of distances between nearest neighbors of vertex *v*. We propose two different approaches to this approximation, hereafter referred to as $d_{ind\_e}$ and $d_{ind\_r}$. Their principles are schematically shown in Fig 4.

**Euclidean distance** of nearest neighbors $d_{ind\_e}$ takes the closest points of the vertex $v\in f_{a}$ from *f*_*i*_ and *f*_*j*_, and computes their Euclidean distance:

$$d_{ind\_e}(v,f_{i},f_{j})=\|near(v,f_{i})-near(v,f_{j})\|$$ (10)

**Relative distance** of nearest neighbors $d_{ind\_r}$ does not use the exact location of the closest points but utilizes their relative direction from $v\in f_{a}$. Relative direction means that either a positive or negative value is used depending on whether the measured surface is in front of or behind the average surface. Being “in front of” or “behind” is delimited by normal vectors assigned to vertices of *f*_*a*_ that have to be properly oriented. If the nearest neighbor is located in the half-space delimited by the normal vector, then the nearest neighbor is considered “in front of” and has a positive distance. And vice versa. Formally,

$$d_{ind\_r}(v,f_{i},f_{j})=\left|d_{r}(v,f_{i})-d_{r}(v,f_{j})\right|$$ (11)

where the relative distance $d_{r}(v,f)$ of the vertex *v* to the face *f* is defined as:

$$d_{r}(v,f)=\left\{ \begin{array}{cc} +\|\vec{v}\| & \text{if \$\textbackslash{}vec\{v}\cdot {\vec{n}}_{v}\geq 0\$\}\\ -\|\vec{v}\| & \text{if \$\textbackslash{}vec\{v}\cdot {\vec{n}}_{v}<0\$\} \end{array}\right.$$ (12)

where $\vec{v}=near(v,f)-v$. ${\vec{n}}_{v}$ denotes for a surface normal at mesh vertex *v*, i.e., the vector interpolating normals of adjacent triangles.

Regardless of the approximation used, the average face *f*_*a*_ serves as a cache that stores precomputed data – either the closest neighbors or their distances. Filling the cache requires searching for the nearest neighbors between the vertices of the average face and all other faces. Therefore, this step is linear with respect to the number of faces in the dataset. Using the cache, i.e., calculating the distances between all pairs of faces via the average face records, remains quadratic. However, this task is very simple and highly parallelizable – the computation of the Euclidean distance in $d_{ind\_e}$ or the relative distance subtraction in $d_{ind\_r}$ can be computed concurrently for all $v\in f_{a}$.

The two approaches differ in specific aspects that may influence computational requirements. The relative distance $d_{ind\_r}$ saves memory because only distances, i.e., single values, are stored for each vertex of the average face. Additionally, it may be faster because the distances to the nearest neighbors are already known from their search. The final distance calculation simply subtracts the values.

In contrast, the $d_{ind\_e}$ method must store the entire 3D coordinates of the closest points. Although the difference in size may seem subtle, comparing an average face consisting of tens of thousands of vertices to hundreds of other faces can lead to high memory demands. Moreover, the final Euclidean distance between two cached nearest neighbors must be computed from scratch, making it slower than simply subtracting two precomputed distances in $d_{ind\_r}$.

Although the relative distance method may seem favorable, its main limitation is that it relies on properly oriented normal vectors of the average face. Moreover, tests performed on real data have shown only negligible differences in speed and precision.

### Auto-cropping

**Fig 5:**
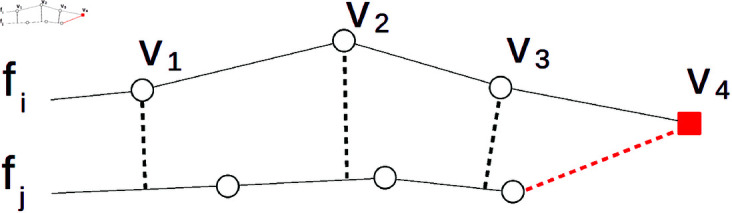
Auto-cropping principle shown in 2D. Dashed lines connect vertices from *f*_*i*_ with their nearest neighbors on *f*_*j*_. The vertex $v_{4}$ of face *f*_*i*_ is detected as lying in the non-overlapping area of *f*_*i*_ since its nearest neighbor is located on the edge of the *f*_*j*_ mesh.

In contrast to point clouds, triangular meshes allow us to easily detect non-overlapping areas by determining whether the found nearest neighbor $near(v,f_{j})$ lies on the boundary edge of the surface. If so, the source point $v\in f_{i}$ lies in the area that does not overlap the other surface and can then be omitted from the computation. This principle is illustrated schematically in Fig 5 showing a cross-section of faces *f*_*i*_ and *f*_*j*_. Also, the black parts of faces D and E in Fig 1 highlight such automatically detected non-overlapping areas of two registered faces.

Omitting non-overlapping areas from the computation helps us tackle two major issues in our approach (*RQ4)*. First, it makes the similarity measurement more meaningful. Non-overlapping areas appear predominantly on the boundaries of facial scans that are not important for identification or face similarity analysis. Even worse, their inclusion in the measurement distorts the results. In contrast, the primary parts of faces, i.e., cheeks, nose, mouth, etc., do overlap. Holes caused by eliminating possibly problematic areas like the beard or folds of the nose also represent non-overlapping surfaces. These areas can then be filtered out using the same auto-cropping mechanism. Therefore, restricting the computation of the distance cost function $d_{avg}$ (Eq 5) or the indirect distance measure function *d*_*ind*_ (Eq 9) to overlapping surfaces ensures that only matching areas are measured, which increases the comparability of obtained distance values.

The second issue is related to the registration phase. As discussed in batch registration, the ICP function suffers from the fact that it is not symmetric. Transforming one face to another can produce different results than inverse superimposition. Therefore, a better direction should be selected to achieve the best precision. However, if ICP is integrated into GPA, then the registration direction is fixed, i.e., faces are always registered onto the template face.

**Fig 6:**
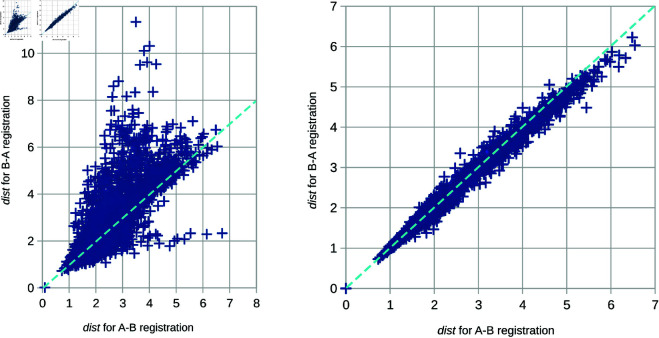
The impact of auto-cropping on the registration. Each cross represents a single pair of faces. The distance function *dist* is computed after registering the first face to the second one (the *x* axis) and vice versa (the *y* axis).

Unfortunately, the asymmetry of the facial stereophotogrammetry data is significant, as shown on the left scatter plot in Fig 6. All pairs from the set of 100 randomly selected faces were registered by ICP in both directions ($f_{i}\to f_{j}$ and $f_{j}\to f_{i}$) using the original distance cost function (Eq 5). Then, their similarity was measured by applying Eq 4. Each point in the scatter plot corresponds to the similarity measurement of one pair. The *x* coordinate shows one direction, while the *y* coordinate shows the inverse direction. The diagonal dashed line delimits perfect symmetry in both directions.

As can be seen in Fig 6, the similarity values deviate significantly from perfect symmetry. The standard deviation between both directions, i.e., the variation of the points on the plot around the dashed line, is 0.65 with a maximum of 7.83.

Podimor *et al*. [40] address the issue of ICP asymmetry by adapting the cost function. Their approach takes into account the nearest neighbors from both surfaces and weights them appropriately so that the pairing is symmetric. However, this solution has a negative impact on efficiency because nearest neighbors in both directions, from *f*_*i*_ to *f*_*t*_ and vice-versa, must be found.

We show that standard ICP can be used for 3D facial scans if the primary source of asymmetry is mitigated at the geometric level, i.e., using a fast auto-cropping function. The right-hand side scatter plot in Fig 6 demonstrates results of the same measurements adapted so that the distance cost function (Eq 5) ignores non-overlapping areas. The standard deviation decreased to 0.07 (maximum 0.96). The Pearson coefficient, which determines the linear correlation between the results from both registration directions, also increased from 0.8143 to 0.9947, proving the significant improvement in the symmetry. Moreover, the average distance of all pairs decreased from 2.37 to 2.34, proving the generic assumption that omitting non-overlapping areas can increase the overall accuracy of ICP [29,30].

These results confirm that the asymmetry in registration and similarity measurement of 3D facial scans is predominantly caused just by the non-overlapping facial areas. Besides possible holes in 3D models, these non-overlapping areas are primarily located on the boundary parts of facial scans. Omitting them makes the computation almost symmetric and then usable for ICP integrated into GPA.

However, limiting ICP to only overlapping parts may not work properly in all situations. For instance, if two faces are positioned so that they overlap only minimally or not at all. In this case, ICP can rely on only limited (or neither) vertices from the overlapping areas and then either converge very slowly or completely fail. Therefore, at least the first ICP iteration should be performed without auto-cropping. However, it is known that ICP is sensitive to the initial position of the surfaces anyway, and some kind of pre-alignment is assumed to get optimal results.

### Optimal sub-sampling

ICP is iterative, taking into account the pairing of many vertices, and is therefore computationally very intensive. As a result, various acceleration techniques are used to reduce computational time. In addition to using space-partitioning structures like k-d trees to search vertices optimally, reducing the number of vertices involved in the computation is another widely used tactic [22,33,34].

Being aware of the specific features of 3D facial scans, where the geometry resembles a shell with scrappy edges rather than obvious enclosed objects, we aimed to research how different sub-sampling methods affect the speed and accuracy of registration (*RQ2*). We tested three sub-sampling algorithms. The random sampling strategy selects *N* random vertices from the original mesh. Uniform sub-sampling uses a 3D uniform grid to cluster mesh vertices into approximately *N* non-empty cells, from which a random vertex is selected. This method ensures a better uniform distribution of selected points. The last method was Gaussian curvature sub-sampling, which takes *N* vertices with the highest Gaussian curvature. They represent significant points on human faces.

The evaluation was performed on a set of 100 randomly selected faces, using the same dataset as for the final evaluation discussed later in this paper. For each face in the set, all other faces were registered towards the face, leading to 4950 face-to-face registrations in total. Each registration was repeated with different sub-sampling strengths, progressively reducing the transformed face from full resolution (approximately 60k vertices) to 50 samples. The first ICP iteration was performed without auto-cropping to ensure sufficient initial alignment. The remaining iterations were done with auto-cropping enabled to get the best matching.

**Fig 7:**
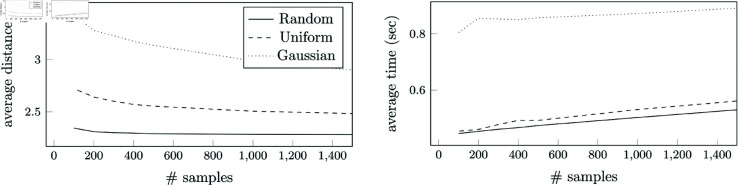
The effect of sub-sampling strategies on registration. The impact on accuracy (left) and efficiency (right) are shown.

The left graph in Fig 7 shows the impact of sub-sampling strategies on accuracy, as measured by the distance function (Eq 4). The *X*-axis captures the number of samples, and the *Y*-axis captures the average distance of the 4950 face-to-face distance measurements. The graph focuses only on the most relevant range, from 50 to 1500 samples, which shows significant trends and correlations between the charts.

Random sampling yields highly precise results, even with extreme sub-sampling down to just a few hundred points. The difference between the full-resolution distance measurement and the 1000-sample measurement was only 0.038 mm. The uniform space sampling performed a little worse and needed thousands of samples to achieve similar accuracy. The results of Gaussian curvature sampling proved unacceptable for our purposes.

The right-hand side efficiency graph in Fig 7 exhibits almost linear acceleration for all algorithms. The fastest registration can be achieved with random sampling, which accelerates the computation from 3000 sec to 0.5 sec (for 1000 samples). The relative slowness of Gaussian curvature sub-sampling is due to the runtime curvature calculation, which can be precomputed. Otherwise, all these methods can be considered similarly efficient for tens or a few thousand samples.

Based on these results, random sampling to 1000 vertices appears to be the optimal setting for batch registration.

### Implementation

The Java implementation of the algorithm can be found in the FIDENTIS Analyst II project https://gitlab.fi.muni.cz/grp-fidentis/analyst2 – an open-source application being developed at Masaryk University for forensic anthropologists. A pseudo-code in Algorithm 1 summarizes all computational phases and optimization tactics. Parameter settings mentioned in the following description were also used for the evaluation.

**Algorithm 1.** Algorithmization of the N:N analysis.



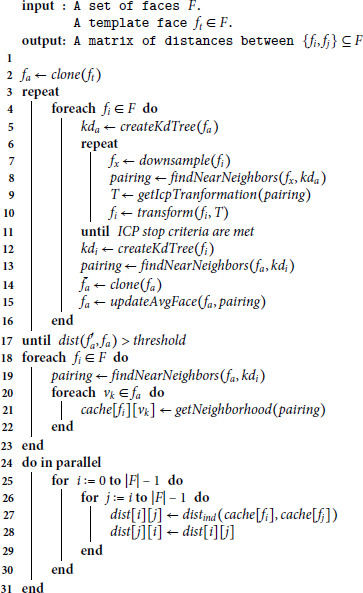



The algorithm takes a set of faces (meshes) as input. One of them is chosen as the template face.

The average face is initialized by cloning the template face (line 2 of the algorithm). The faces are then repeatedly registered against the average face until the average face stabilizes (lines 3–17). We use the *Procrustes Surface Metric*, as defined in [40], with a threshold of 0.3 as the termination criterion for the GPA on line 17.

As a stopping criterion for ICP iterations on line 11, we use the combination of a maximal number of iterations (set to 100) and accuracy change. The latter checks the difference between distances $d_{avg}(f_{a},f_{i})$ computed in two consecutive iterations. If the difference is less than 0.05, then the ICP registration of the face terminates. We observed that 3–9 iterations are typically required to reach this threshold.

K-d trees are used to accelerate the search for the nearest neighbors in the ICP [23,24]. The construction of a k-d tree for a specific face is suggested by the *createKdTree* function in the pseudo-code. The utilization of the k-d tree for the nearest neighbor search is provided by the *findNearNeighbor* invocation, which takes a mesh of a source face as the first argument and the mesh stored in a k-d tree as the second argument. For each vertex of the source face, the function finds the closest point on the second face using a fast k-d tree search. The *pairing* output encodes the final mapping – for each vertex of the first face, we know its closest point from the second face. The obtained pairing is then used for the computation of the transformation matrix and to update the shape of the average face by the *updateAvgFace*() function. In accordance with Eq 2, vertices with multiple nearest neighbors are excluded from the pairing. Moreover, except for the very first ICP iteration, vertices from non-overlapping areas are also excluded. The first iteration uses all vertices to ensure correct superimposition for faces with minimal or no initial overlapping.

We use random sub-sampling with 1000 vertices to implement the *downsample*() function on line 7.

The N:N distance measurement is divided into two parts. First, the closest neighborhood of all faces is computed for each vertex of the average face (lines 18–23 of the algorithm) and stored in the *cache*. What data is stored depends on the preferred indirect distance (Eq 9). The cached values are then used to compute the real distance between all pairs of faces from the dataset (lines 24–31). This is the only part with quadratic complexity (concerning the number of faces). However, as the final computations (lines 27–28) are simple and independent, they can be solved quickly in parallel.

## Results

While the evaluation of the effect of cropping and sub-sampling on the registration and measurement was made as part of the algorithm’s design decisions, this section focuses on proving the usability of the final proposed algorithm for fast yet precise N:N analysis.

### Data

3D facial scans used for the evaluation were obtained from the FIDENTIS 3D Face Database [43]. Faces were recorded with a stereophotogrammetric-based Vectra M1 3D facial scanner. The acquired 3D images were subsequently processed in accordance with the protocol as stated in [43]. We used the *_ECA* version of models for the evaluation, i.e., edited uniformly trimmed facial scans encompassing frontal ear-less parts of the face and possibly holes as the result of data preprocessing occasionally.

We used three datasets consisting of 100, 500, and 1000 faces, hereafter referred to as *D*_1_, *D*_2_, and *D*_3_. *D*_3_ included the first 1000 faces from the database and covered different genders and ages. The smaller dataset *D*_2_ was created as a subset of randomly selected faces from *D*_3_. Both *D*_3_ and *D*_2_ were used only for efficiency tests. Faces for the *D*_1_ dataset were selected randomly from *D*_2_ and used for time-demanding computations, especially the accuracy evaluation. The variability of mesh resolutions (number of vertices) in all datasets is summarized in Table 1.

**Table 1:** Size of datasets (number of faces) and the resolution of scans (min/max/avg/median number of vertices).

dataset	# faces	min.	max.	avg.	median
*D*_1_	100	29,205	75,841	56,680	58,184
*D*_2_	500	17,240	212,945	54,468	57,861
*D*_3_	1000	14,382	212,945	54,087	57,778

### Test configurations

To evaluate the efficiency and accuracy of our solution, we compared four different variants of N:N registration and similarity measurements. They reflect different levels of efficiency optimization, allowing us to assess the potential errors introduced by our solution.

**Baseline (BL)** algorithm provides a slow but highly precise computation, which can be considered a standard anthropologist approach. It follows the routine analytical workflow, in which standard IPC-based registration and face-to-face measurements are applied to all pairs in the dataset to achieve the highest level of precision. Each pair of faces is registered individually in both directions ($F_{i}\to F_{j}$ and $F_{j}\to F_{i}$), considering only the best result (i.e., the direction with a smaller distance). Faces are registered at full resolution (i.e., without sub-sampling) using the auto-cropping functionality after the first ICP iteration. Also, the similarity measurement is conducted precisely by computing the direct distances $d_{avg}$ between all pairs individually, using auto-cropping. This method serves as the baseline solution for the comparison with other (faster) methods as it ensures the most precise measurement with no optimizations.

**Fast registration (FR)** algorithm combines our fast linear-time registration with the slow but precise pairwise measurement used in BL. The adapted Procrustes approach with ICP registration and random sub-sampling to 1000 vertices is used to align all the faces towards an averaged template face iteratively. Subsequent similarity measurement is conducted for each pair individually, as in the BL approach. Auto-cropping is used for both registration and distance measurement. This configuration aims to assess the impact of our fast but approximate batch registration on the overall time requirements and, in particular, on accuracy.

**Indirect vector distance (IVD)** approach represents a complete implementation of the Algorithm 1. Euclidean distance $d_{ind\_e}$ is used for distance caching and similarity measurement. Therefore, this algorithm functions similarly to the FR version. However, instead of comparing all pairs of registered faces, the average face is used as a gauge for indirect distance calculation.

**Indirect relative distance (IRD)** algorithm operates similarly to IVD, but relative distance $d_{ind\_r}$ is used for distance caching and measurement.

### Efficiency

That the goal of reducing quadratic complexity has been achieved is evident from the algorithmization. Table 2 demonstrates the impact of nearly linear time computation on practical usability. The times for the baseline *BL* method should be considered informative only as the goal of this configuration was to achieve the highest precision regardless of efficiency. Nevertheless, the results of the *Fast Registration* method indicate that accelerating the registration phase solely by combining the Procrustes method with ICP and sub-sampling does not yield significant improvements, as the pairwise measurement still poses a significant bottleneck. Only the combination of fast registration with indirect measurement tackles time complexity and enhances scalability.

**Table 2:** Time requirements (hh:mm) of N:N processing methods.

dataset	# faces	BL	FR	IVD	IRD
*D*_1_	100	13:07	03:05	00:11	00:11
*D*_2_	500	>24h	>24h	00:55	00:54
*D*_3_	1000	>24h	>24h	02:03	02:02

Another important observation from efficiency tests is that the difference in speed between the IRD and IVD methods is negligible. The choice of the indirect method does not significantly affect overall efficiency.

The evaluation was conducted on a laptop with 4 CPU cores, 1.8 GHz, and 16 GB of RAM. Long-lasting experiments were terminated after 24 hours of computation. Due to the time requirements of experiments, methods dealing with an average face, i.e., FR, IVD, and IRD, were performed with five different initial template faces, and then the time was averaged. Hereafter, these faces are denoted as *A*,*B*,*C*,*D*,*E*. Although they were selected randomly, they included different genders and ages to cover different shapes and sizes of mesh models.

### Accuracy of registration

Our fast registration is approximate, which introduces a certain degree of imprecision. In particular, the imprecision arises from three factors: all faces are superimposed towards a single common average face, the standard cost function is used inside ICP with auto-cropping, and faces are sub-sampled. This evaluation aims to study errors introduced by these three factors by assessing how precisely the results of our approximate registration oscillate around the values produced by the baseline method.

**Fig 8:**
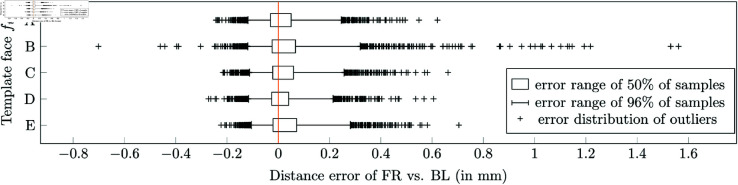
Accuracy of fast registration. Distribution of distance errors introduced by our fast registration (FR) to the baseline algorithm (BL). Results of five different template faces A–E are shown. 0 = the same results, positive error = BL registered more precisely, negative error = FR registered more precisely.

Fig 8 compares the accuracy of BL and FR utilizing five distinct template faces *A*,*B*,*C*,*D*,*E*. The *X* axis represents errors computed as the difference between the similarity measurements returned by the FR and BL methods. Zero means identical measurements. Positive numbers indicate imprecision introduced by FR. Negative values increase precision introduced by FR. Errors were calculated for all pairs of faces in the *D*_1_ dataset. The distribution of errors is captured by the box plot for 2nd–25th–75th–98th percentiles. It means that the rectangular parts cover 50% of samples (from 25% to 75%), thin lines the lower and upper 23% (96% of all samples), and the remaining upper and lower 2% of outliers (4% at all) are depicted explicitly as crosses.

The box plots show that the majority of faces are registered very closely to the results obtained by the baseline algorithm. The maximal error of 50% of face pairs (the rectangular parts on the box plot) is less than 0.07 mm, and the maximal error of 96% of pairs (the thin lines including the rectangular parts) is less than 0.3 mm.

An in-depth analysis of the data revealed that facial similarities computed by the baseline method range from 0.72 to 6.22 mm, with an average of 2.34, where zero indicates perfectly identical facial scans and larger values indicate greater dissimilarity between faces. Taking into account this distribution, the error introduced by the FR method, i.e., up to 0.07 mm in 50% of cases and 0.3 mm in 96% of cases, can be considered marginal. Moreover, the results do not vary significantly between template faces *A*–*E*. Nevertheless, the face *B* demonstrates that the selection of an inappropriate template face can have a certain negative effect on results and that appropriate expertise in anthropometry is required when selecting the initial template face.

### Accuracy of similarity measurement

Indirect similarity measurement can introduce another degree of imprecision into the N:N analytical workflow. In particular, the imprecision arises from replacing two-directional vertex-to-nearest-neighbor distance measurements with approximate $d_{ind\_e}$ or $d_{ind\_r}$ methods.

**Fig 9:**
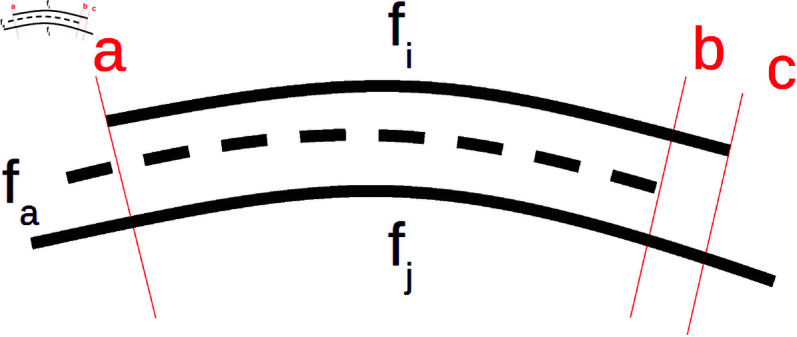
The impact of auto-cropping on direct and indirect surface measurement. The overlapping area cropped to all three faces (defined by cutting edges *a* and *b*) is smaller than the combined area of faces *f*_*i*_, *f*_*j*_ excluding the average face *f*_*a*_ (cutting edges *a* and *c*).

To analyze the possible errors introduced by the measuring optimizations, we compare IRD and IVD with FR. However, we must account for the different surface areas involved in the calculation of IRD/IVD and FR due to auto-cropping. The problem is illustrated in 2D in Fig 9. The solid lines represent cross-sections of two faces *f*_*i*_ and *f*_*j*_ that are to be measured, and the dashed line is the average face *f*_*a*_. The cutting edge *a* demonstrates the situation when there is no difference between overlapping areas of $f_{a},f_{i}$, and *f*_*j*_ used by IRD/IVD, and $f_{i},f_{j}$ used by FR. However, if $f_{i},f_{j}$ extends beyond the *f*_*a*_, then the overlapping areas used by IRD/IVD and FR differ (cutting edges *b* and *c*).

As the FR and IRD/IVD measure different surfaces, their direct comparison is misleading. Therefore, we adapted FR for this evaluation so that the surfaces are cropped to the average surface, even though the average surface is not involved in the distance computation itself. The experiments were conducted on the same dataset and with the same template faces (*A*,*B*,*C*,*D*,*E*) used in the registration accuracy evaluation.

**Fig 10:**
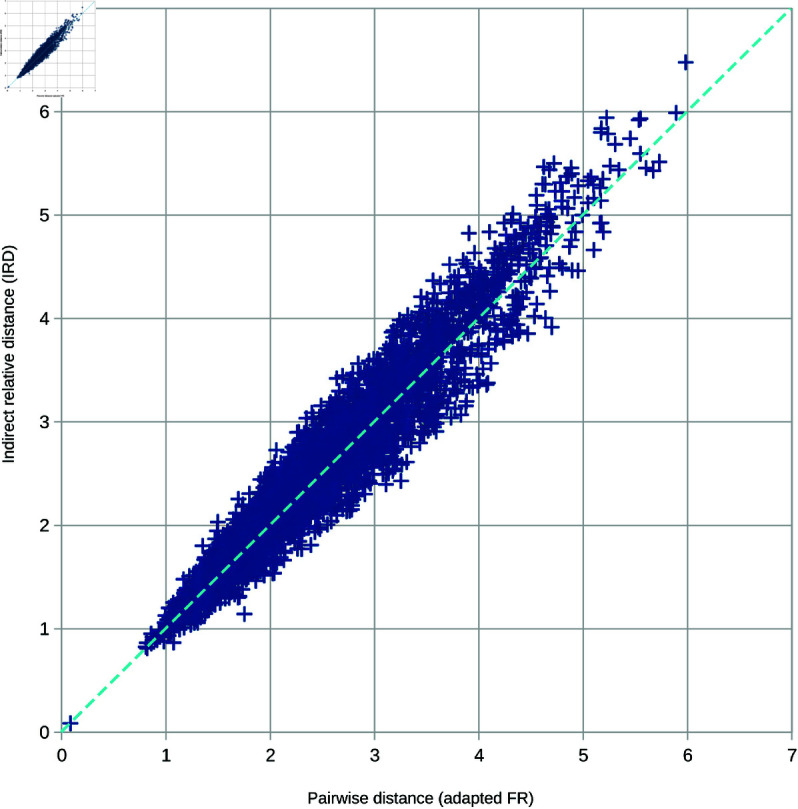
Correlation of the direct and indirect measurement. The scatter plot captures the differences between slow pairwise distance measurement (the *x* axis) and fast indirect relative distance measurement (the *y* axis).

The scatter plot in Fig 10 shows the difference between the adapted FR and IRD. Each cross represents a pair of faces. The results of the adapted FR measurement are captured on the *X* axis, while the *Y* axis captures the results of fast IRD. The closer the cross is to the diagonal, the smaller the difference is. Face *A* was used as the template face for this graph. However, other template faces exhibit very similar distributions.

The standard deviation of error between both methods is only 0.15 mm with a maximum of 0.92 mm. The Pearson correlation coefficient of 0.9629 further confirms a substantial similarity between both approaches, indicating that direct FR-based measurement and indirect IRD-based measurements generate very similar values. We also observed that the differences between IRD and IVD are negligible.

### Accuracy of the whole algorithm

The previous experiments aimed to analyze errors that may have been introduced separately in the registration and measurement processes. They provide insight into the accuracy of the two steps, which are related but relatively independent. However, these isolated results do not fully reflect the overall accuracy of our entire algorithm, as the applied simplifications could influence each other either positively (suppressing the error) or negatively (increasing the error).

Unfortunately, directly comparing the absolute values produced by the baseline and our optimized methods is misleading due to the different (auto-cropped) surfaces involved in the computation. This issue of comparing BL with IRD/IVD is similar to the FR vs. IRD/IVD comparison discussed in the previous section. Auto-cropping used in BL often involves larger areas, as only two faces are always registered and measured at a time. In contrast, IRD/IVD involves smaller areas as all faces are cropped to the common average face. However, in this case, it is impossible to unify the result by cropping faces to the average face because the baseline method does not use the average face at all. It is also difficult to decide whether dealing with the larger or smaller areas is better or worse from the perspective of anthropometry. A smaller area focuses on important central parts of a face, possibly omitting some significant peripheral areas like ears. Conversely, larger areas may involve undesirable peripheral noisy parts in calculations.

Therefore, instead of comparing absolute similarity values produced by respective methods for each facial pair, we focus on the higher-level analysis – the ability of the methods to identify the same clusters of (dis)similar faces. Clustering represents a key use case of the N:N analysis and is crucial for studying human variations by anthropologists.

Statistical analysis of BL and IRD/IVD methods applied to the *D*1 dataset proved a significant correlation. The Pearson coefficient of 0.9997 for face *A* proves that both methods have the same overall ability to distinguish similar and dissimilar faces.

A comprehensive assessment of clustering abilities would require defining specific anthropological scenarios, hypotheses, and clustering methods, which is beyond the scope of this paper. However, since humans excel at recognizing visual patterns, we conducted a simple qualitative experiment. We transformed the distance values measured by BL and IRD/IVD into heatmaps – a widely used visual analysis technique employed by domain experts for interactive exploration of clusters [44,45].

**Fig 11:**
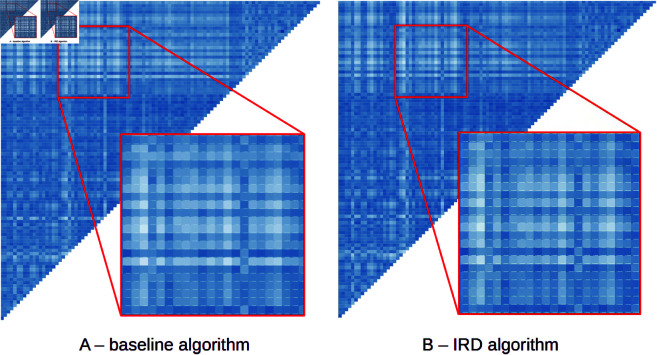
Comparison of distance heatmaps. Visually similar heatmaps with almost identical color patterns indicate similar clustering abilities of both approaches. The saturation of each cell reflects the measured distance between the face pairs. The left heatmap was generated by the baseline algorithm, while the right one was produced by our fast IRD algorithm.

Heatmaps for the dataset *D*_1_ are depicted in Fig 11. Each cell in the color half-matrix represents a distance of two faces transformed to a color scale. The darker colors encode smaller distance values (more similar faces) and vice versa. The left heatmap encodes the precise BL-based pairwise measurement, while the right heatmap is for IRD using the face *A* as a template face.

Both heatmaps are visually almost identical, with the same color patterns recognizable by the analysts. Note that the goal of this experiment was not to evaluate the correctness of visual patterns, only their similarity. The ability to present the same similarity patterns in the heatmap confirms the strong correlation results of both methods and that the clustering capabilities of IRD/IVD have been preserved. The comparison of heatmaps for template faces *B* – *E* produced the same results.

Both heatmaps appear visually almost identical, with the same color patterns recognizable by sight. It is important to note that the goal of this experiment was not to assess the correctness of the visual patterns but rather their similarity. The ability to present consistent patterns with no evident discrepancies confirms that the clustering capabilities of IRD/IVD have been preserved. A comparison of heatmaps generated for template faces *B*–*E* yielded the same results.

## Conclusions

Our algorithm advances the field by providing a fast and accurate solution to a scenario where an expert requests a large-scale preliminary search of the 3D dataset of polygonal facial scans to find and/or group the most suitable candidates, which can then be subjected to more in-depth testing and quantification of the degree of similarity or differences. Our approach will register facial scans and provide a simple quantification of (dis)similarity for comparison/grouping.

### Addressing the research questions

This paper aimed to answer four research questions.

*RQ1: How can a set of polygonal 3D facial scans be registered efficiently, yet precisely?* The linear complexity of batch registration was achieved by carefully combining existing approaches and utilizing features of facial 3D scans. We have shown that generalized Procrustes superimposition can be combined with one-directional ICP if the asymmetry is suppressed by automatically cutting out non-overlapping parts of meshes. Sub-sampling can further accelerate the process without decreasing accuracy.

*RQ2: How do the sub-sampling methods affect the efficiency and precision of batch registration?* We evaluated the impact of three distinct sub-sampling strategies on the registration. Random sampling appears to enable us to reduce the number of points radically, from tens of thousands to hundreds, still preserving sufficient speed and precision. Therefore, we used random sampling with a reduction of 1000 samples during evaluation experiments. However, other sub-sampling strategies could be used as well. Their impact on the overall performance should be rather subtle if they are reasonably adjusted.

*RQ3: How to measure a mutual similarity of a set of registered 3D facial models efficiently?* By reusing the computed average face as a gauge with pre-cached values, we reduced quadratic complexity to near-linear time. We have shown that the possible error introduced by approximate indirect measurement is marginal.

*RQ4: How to deal with holes and scrappy edges?* Polygonal meshes enable us to detect non-overlapping surfaces very quickly. We have shown that both the registration and measurement phases can benefit from the introduced auto-cropping mechanism without the loss of efficiency.

### Limitations

Our solution was developed and tested on facial scans. Other 3D models often used in studies of human biological distances, such as crania, might not produce sufficiently precise results with the presented parameters due to their more complex geometry. Although our algorithm is designed to be generic, further research is needed to determine its applicability to geometries with significantly different characteristics.

Occlusion, different head poses, or facial expressions that pose challenging issues in 3D facial identification are not addressed by our approach, either. However, the presented algorithm can serve as a generic framework in which the cropping and distance measurement steps can be replaced with more advanced methods tailored to handle these issues.

The proposed algorithm strongly depends on polygonal meshes. 3D scans captured as point clouds must be converted into meshes in preprocessing, which can be laborious. Also, we suppose reasonably dense and uniformly distributed mesh vertices. Significant differences in densities of mesh models or significantly uneven distribution of vertices can introduce asymmetry into the registration and measurement, possibly negatively affecting the precision of results. In this case, the registration would have to use a more expensive bidirectional measurement like in [40]. In general, the impact of the mesh properties on the indirect measurement remains an open question.

### Future work

Our research aimed to propose an efficient yet generic algorithm for the clustering of facial scans. However, the intentions of anthropologists are usually specific, e.g., assessing similarities and dissimilarities between facial features in order to assess their biological distance. However, it appears that various data collections can differ in the ability to identify clusters at all, e.g., collections of adults vs. children. Therefore, the usability of our generic approach for the pre-selection of faces from such specific datasets and for specific analytical intents will be researched in the future.

Currently, the selection of a template face for alignment and gauge-based measurement is left to the expertise of biological anthropologists. A fully automated process would select (or at least advise) a face that is most similar to all other faces in the dataset. Unfortunately, this computation again introduces quadratic complexity. Addressing this challenge will be a key focus of our future research.

The registration phase can produce insufficient results if the faces are not pre-aligned. Although this is the generic drawback of the ICP algorithm, which is not specific to our solution, fast pre-alignment strategies for batch processing have to be invented and integrated into the batch process to make it more robust. For 3D facial scans, bounding boxes or symmetry planes [46] could be used for this purpose. Another solution would be to utilize AI to estimate significant landmarks quickly, though not reliably, and superimpose them approximately using the standard fast Procrustes approach.
